# Mental health services in new Libya: the way forward

**DOI:** 10.3402/ljm.v8i0.21581

**Published:** 2013-07-22

**Authors:** Selim M. El-Badri

**Affiliations:** Waikato District Health Board, Hamilton, New Zealand. Email: elbadrism@gmail.com

The general health system in Libya has been marred by many systemic problems, most notably inadequate mental health services (1–4). Psychiatric services are hospital-based and are mostly centralised in major cities, with underdeveloped community and specialised services. Post-war reports predicted gloomy prospects and indicated that thousands of people in Libya will need treatment for mental health problems (5). Many mental health workers and members of voluntary organisations agreed that it is time to make a change and establish a much more suitable, culturally sensitive, accountable, and responsive mental health service in Libya. Modern facilities with adequate staffing and resources are required to facilitate the shift towards patient-oriented community-based services. The drive to create this collaborative approach is consistent with Libyan cultural and Islamic values which consider the mentally ill to be vulnerable and deserving of humane treatment and protection.

Strategic planning should consider moving mental health services towards a more inclusive and participative model of health care. Mental health care should be provided through general health services and community settings (6). Large, centralized, and isolated psychiatric hospitals are not sensible and need to be replaced by other more appropriate mental health services. The current services in Libya continue to provide long-stay custodial care for many patients regardless of the nature of their mental illness. This type of care is not justified by its cost, effectiveness, or the quality of care provided. If there is a need for long-stay facilities for some patients, supported accommodation in small units or houses in the community should be considered. Integration of mental health services into general health services is required to overcome segregation and associated stigma. This will facilitate early identification and treatment of mental disorders and accessibility to medical care and related facilities.

The main clinical focus of in-patient services should be on the short-term stabilisation of acute psychiatric disorders, emphasising discharge and follow-up planning to help patients move quickly to less restrictive alternative care. Provision for and consideration of alternative care for related problems, such as detoxification services to substance abusers, as well as dual-diagnosis programming, should be considered and developed accordingly.

Some of the service development priorities should also be the development of specialised mental health services (e.g. child and youth, forensic, old age) as well as the development of effective mental health promotion, prevention, and early intervention strategies. The success of this will depend on the enhancement of training and support for staff and other service providers.

A case management approach is essential, with a multidisciplinary team typically consists of at least one psychiatrist, a mental health nurse, occupational therapist, a psychologist or social worker (7). One of the case manager's main roles is to work within and ensure that an integrated programme of health care and social support for the patient is in place. The aim should be to help the patient gradually achieve recovery of social, occupational and general functioning and not just symptomatic remission.

Good continuity of outpatient care should be the norm to achieve and maintain desired clinical outcomes. Patients should be supported by staff who are familiar to them and with whom they have a continuous relationship. Applying the catchment area method of organising services is a well-established approach to resolving the need for continuity of care (6).

Services should respect the autonomy of patients with mental illness, should empower them to make informed decisions and choices about their treatment, and should use the least restrictive types of treatment. Human rights should be considered at an early stage of service development and implementation, monitoring and evaluation of mental health policies and programmes in new Libya.

Health care providers should be interested in knowing whether their patients have received the programme of health and social support according to their assessment recommendations and whether targets have been met. The use of outcome measurements can help people with their progress and assist with providing early intervention and help services to collect data which can support service development, research and quality improvements. Data collection and analysis can be facilitated by the creation of active and progressive audit and clinical governance systems that can be linked to medical informatics and technology networks. This should also link in with a local, regional, and national statistics body. It is equally important to continue to monitor the mental health care outcome measurement literature and make appropriate recommendations in due course.

There is a need to create a Mental Health Commission (MHC) that should prepare a Strategic Plan for the mental health system in consultation with providers of mental health, and after reviewing the current service. Some anticipated key roles of the committee are described as follows:

1. to review different perspectives of service delivery and resources including inpatient and community care and related provisions;
2. to review models of best practice in the current environment of recovery-oriented mental health services and to define what would work best for the country.
3. to consider how mental health services in Libya could incorporate the recovery model into service delivery and what is required to ensure its successful implementation.
4. to consult with and seek input from people with mental illness and also their families and expertise and personnel working in the mental health services in Libya and worldwide (e.g. WHO), to ascertain the extent to which such changes to service delivery and model of care should be considered and incorporated into the Libyan mental health services.

Psychiatric stigmatisation remains a fundamental issue worldwide (8) and Libya is no exception. However, in Libya, as in many other countries, there is no legislation covering discrimination against mentally ill people. Also, there is no Mental Health Act to regulate, for example, the grounds under which a mentally ill person can be detained or treated. The establishment of a Libyan MHC would be a step in the right direction.

In summary, a proposal drawing on a variety of international principles, standards, and experience can be adopted and modified if necessary to suit the Libyan circumstances. An MHC could be charged with preparing a plan to chart the way forward.

*Selim M. El-Badri* Waikato District Health Board Hamilton, New Zealand Email: elbadrism@gmail.com
